# Health service delivery and disease management across Ethiopian health care facilities: An evidence-based analysis of tuberculosis program performance and system determinants

**DOI:** 10.1016/j.dialog.2026.100322

**Published:** 2026-06-02

**Authors:** Dagmawi Mengesha

**Affiliations:** School of Medical Laboratory Science education and services, College of Medicine and Health Sciences, Bahir Dar University, Bahir Dar, Ethiopia

**Keywords:** Tuberculosis, Health service delivery, Ethiopia, Implementation science, Primary care

## Abstract

**Background:**

Ethiopia's health system faces a triple burden of infectious diseases, notably tuberculosis (TB), non-communicable diseases (NCDs), and injuries, challenging service delivery in resource-limited settings.

**Methods:**

We conducted a mixed-methods analysis of TB program performance and health system determinants across 112 Ethiopian health care facilities using a historical baseline dataset (2010−2011). We integrated quantitative data (spatial analysis and mixed-effects logistic regression) with qualitative insights (policy analysis and stakeholder interviews), adhering to RECORD and COREQ guidelines.

**Result:**

Pediatric TB detection was critically low (2.1% vs. WHO's 12%), and treatment success varied significantly by facility type (hospitals: 88.2%; primary health care units [PHCUs]: 63.8%; chi^2 = 34.2, *p* < 0.001). Key system bottlenecks included low staff density (0.7/1000) and supply chain interruptions strongly correlated with treatment failure (*r* = 0.72). Qualitative triangulation revealed that diagnostic uncertainty and irregular supervision directly compounded these institutional gaps.

**Interpretation:**

We propose a “Five-Pillar Framework” diagnostic strengthening, community systems, supply chain reform, digital health, and governance improvements to advance universal health coverage (UHC). While based on foundational baseline data, these findings offer a transferable model for low- and middle-income countries (LMICs).

## 1 Introduction

Ethiopia, with a population exceeding 120 million, confronts a complex epidemiological transition, balancing persistent infectious diseases such as tuberculosis (TB; 4% of Africa's burden) against rising non-communicable diseases (NCDs) driven by urbanization and aging [1], [2]. Ethiopia now faces a “triple burden” of disease: infectious conditions like TB, rising NCDs, and a growing burden of injuries [3], [4]. This amplifies strain on a system already challenged by low workforce density (0.7/1000), chronic supply chain disruptions, and stark urban-rural inequities [5], [6], [7]. TB, with an incidence of 140 per 100,000, exemplifies these system-wide challenges [1], [8]. (See Table 1, Table 2.)

**Table 1:** TB treatment outcomes by facility type.

Facility Type	Case Contribution	Treatment Success Rate (95% CI)
Referral/General Hospitals	39.3%	88.2% (84.1–91.5%)
Health Centers	38.7%	76.5% (71.8–80.7%)
Primary Health Care Units (PHCUs)	22.0%	63.8% (51.3–74.9%)

Note: Differences in success rates across tiers were highly significant (chi^2 = 34.2, p < 0.001) [9].

**Table 2:** Predictors of TB treatment success (mixed-effects model).

Predictor	Adjusted Odds Ratio (AOR)	95% CI	*p*-value
Facility Type *(Ref: PHCU)*			
Referral/General Hospitals	2.1	1.4–3.2	< 0.01
Health Centers	1.6	1.1–2.4	< 0.05
Staff Density *(per 0.1 increase)*	1.8	1.1–2.9	< 0.05
Stockout Duration *(per week)*	0.9	0.85–0.95	< 0.01

To address these disparities, Ethiopia launched the Health Extension Program (HEP) in 2003. The HEP is a flagship community-based primary care initiative deploying Health Extension Workers (HEWs) to rural *kebeles* to provide basic curative and preventive services. Evidence suggests the HEP has expanded primary care access to over 85% of rural Ethiopians [12], [13].

The limitations of community-based frameworks in managing complex, high-burden infectious diseases are well-documented across sub-Saharan Africa. Studies evaluating task-shifting models show that while community health workers excel at preventive education, their clinical impact is frequently truncated by systemic under-resourcing [33]. Specifically, diagnostic delays driven by a lack of essential laboratory infrastructure and irregular, non-supportive supervision have been shown to severely compromise pediatric case detection and treatment compliance in comparable low- and middle-income country (LMIC) settings [34]. By failing to reinforce primary tiers with robust referral and diagnostic loops, health systems inadvertently concentrate successful outcomes in tertiary centers, a phenomenon observed across East African health systems facing similar structural constraints.

This study examines TB program performance and health system determinants across Ethiopian primary health care facilities, including General/Referral Hospitals, Health Centers, and Primary Health Care Units (PHCUs) [9], [10]. We utilize a comprehensive dataset from 2010 to 2011. While historical, this period serves as a crucial baseline to identify persistent systemic bottlenecks such as staffing ratios and supply chain mechanics that predate recent interventions like GeneXpert, offering vital lessons for current health policy. To evaluate these dynamics, we employ three frameworks: Complex Adaptive Systems Theory, the Socio-Ecological Model, and Implementation Science Principles [11], [12], [13], [14].

### 1.1 Objectives

1. To quantify TB outcomes across facility types;
2. To identify health system bottlenecks using data triangulation; and.
3. To propose a “Five-Pillar Framework” for scalable strategies aligned with the WHO End TB Strategy and SDG 3.8.

## 2 Methods

### 2.1 Study design and setting

We conducted a mixed-methods study across 112 facilities (20 referral hospitals, 25 general hospitals, 45 health centers, 22 PHCUs) in five Ethiopian regions, selected via stratified random sampling. The reporting follows STROBE, RECORD, and COREQ guidelines [15], [16].

### 2.2 Data sources

#### 2.2.1 Quantitative

TB case notifications and system indicators were extracted from the District Health Information System (DHIS2) and facility registers (TB registers, stock cards) for the period January 2010 to December 2011 [10].

#### 2.2.2 Qualitative

We analyzed 15 policy documents and conducted 10 semi-structured interviews with stakeholders (5 HEWs, 3 health officers, 2 zonal TB coordinators) selected via purposive maximum variation sampling [16].

#### 2.2.3 Data quality

The WHO's Data Quality Review toolkit confirmed completeness (98.2%) and consistency (kappa = 0.87, *p* < 0.001) [17].

#### 2.2.4 Statistical analysis

We used descriptive statistics and Moran's I for spatial clustering (alpha = 0.05) [8]. To account for the hierarchical nature of the data and spatial clustering, we employed a mixed-effects logistic regression model (with facilities as random effects) to predict treatment success based on facility type, staff density, and stockout duration [9]. Qualitative data underwent framework thematic analysis, which was systematically integrated into the result areas to supplement the quantitative metrics [15].

#### 2.2.5 Ethics and data governance

The original data collection was conducted under routine FMOH reporting mandates. Ethical clearance for the secondary analysis, de-identification, and publication of this dataset was granted by the Ethics Committee in January 2025 (Ref: ETH-2025-001). All data were fully anonymized prior to analysis.

## 3 Results

### 3.1 Case notification, detection disparities, and diagnostic uncertainty

Of the 876 TB cases evaluated, 62.4% were male (chi^2 = 12.3, *p* < 0.01) [18]. A stark disparity was observed in pediatric cases (0–14 years), which accounted for only 2.1% of the total caseload [19]. This is significantly lower than the WHO national estimate of 12%, signaling substantial under-detection in primary care tiers [1].

Our qualitative triangulation directly illuminates the drivers behind this missing cohort. Thematic analysis revealed that diagnostic uncertainty acts as a primary bottleneck at the grassroots level [15]. Because primary health facilities and community posts frequently lack laboratory infrastructure, HEWs are forced to rely heavily on clinical signs that overlap with other childhood illnesses [20]. As one HEW respondent noted: *“We do not have sputum microscopy or x-rays here. When a child presents with a chronic cough, it is highly challenging to separate severe malnutrition or common pneumonia from tuberculosis without diagnostic confirmation. We must rely purely on our clinical judgment.”*

### 3.2 Institutional treatment outcomes and supply chain bottlenecks

Quantitative analysis demonstrated that TB treatment outcomes are heavily dependent on the institutional tier [9]. Referral and general hospitals handled the largest share of cases and achieved the highest treatment success rates, while primary health care units (PHCUs) lagged substantially.

To understand the systemic drivers of these disparities, we analyzed human resource and logistical variables [6], [7]. The median staff density across the sample was 0.7 per 1000 population, falling far short of the WHO benchmark of 2.3 per 1000 [5], [6]. Furthermore, stockouts of first-line TB medications were strongly correlated with treatment interruptions (*r* = 0.72, *p* < 0.05) [7], [21].

When adjusting for confounding and accounting for institutional clustering, the mixed-effects logistic regression model confirmed that facility infrastructure, staffing levels, and medication continuity are independent predictors of patient recovery [9].

This model establishes that treatment within an urban referral or general hospital is associated with more than twice the odds of success compared to a PHCU. Crucially, for every additional week of drug stockout experienced by a facility, the individual patient's odds of treatment success drop by 10% (AOR: 0.9).

Our qualitative interviews provide critical context for these statistical associations, highlighting two interconnected systemic breakdowns [16]:

#### 3.2.1 Supply chain unpredictability

Quantitative stockout durations directly manifest as broken patient trust and subsequent default [21]. An HEW respondent summarized this breakdown clearly:

*“When drugs run out, patients lose trust and don't return. They travel long distances over difficult terrain to reach us; if we send them away empty-handed, they lose faith in the system and stop taking their medications entirely.”*

#### 3.2.2 Fragmented supervision

The low performance of PHCUs is further explained by a severe deficit in programmatic oversight, with only 35% of facilities receiving their mandated quarterly supervisory visits from zonal or regional coordinators [22]. This structural neglect leaves rural staff isolated, unable to troubleshoot stock shortages or resolve complex clinical management issues in a timely manner [25].

## 4 Discussion

This study highlights critical, structural gaps in the Ethiopian health system using a comprehensive baseline dataset [10]. The 24.4 percentage point gap in treatment success between hospitals (88.2%) and PHCUs (63.8%) strongly underscores the “inverse care law,” where rural, underserved populations receive lower quality care despite bearing a heavy portion of the disease burden [11].

### 4.1 Interpretation of findings through theoretical frameworks

#### 4.1.1 Diagnostic gaps and structural biases

The 49.4% prevalence of Extrapulmonary TB (EPTB) within this cohort significantly exceeds historical Federal Ministry of Health (FMOH) norms of 35–40% [22]. In a high-quality health system, elevated EPTB requires robust bacteriological confirmation [11]. However, our integrated results suggest this spike is likely an artifact of diagnostic over-reliance on clinical signs due to the absence of reliable microscopy or molecular tests [23]. This clinical uncertainty directly mirrors the severe under-detection of pediatric TB (2.1% observed vs. 12% expected) [19], [24]. Missing pediatric cases are a direct systemic symptom of rural infrastructure deficits, matching broader regional patterns of missed childhood diagnoses [24]. This baseline reality aligns precisely with the demographic realities quantified in the national census surveys of the same baseline era [36].

#### 4.1.2 Complex adaptive systems and supervision failures

Viewed through Complex Adaptive Systems Theory, a health system relies on tightly coupled feedback loops to maintain performance [12]. The low median staff density (0.7/1000) coupled with a 35% routine supervision rate ruptures these feedback loops [6], [25]. When primary healthcare workers are structurally isolated and overwhelmed by clinical volume, their capacity for active community case-finding and tracing defaults collapses [20]. This dynamic illustrates how macro-level resource constraints yield micro-level program failures, matching systemic behavioral assessments in East Africa [37].

#### 4.1.3 Socio-ecological impacts of supply chain failure

Our mixed-effects regression proves that a one-week stockout decreases the odds of treatment success by 10%. Applying the Socio-Ecological Model demonstrates that supply chain failure is not merely a logistics problem, but an institutional shock that reshapes individual patient behavior [13]. When the institutional level fails to guarantee medication availability, it erodes patient trust, alters community perception of health service efficacy, and directly drives treatment interruptions and drug resistance [21].

### 4.2 Policy relevance and the post-GeneXpert landscape

While these data represent a historical baseline (2010–2011), they capture foundational vulnerabilities that predate the large-scale rollout of contemporary tools like GeneXpert and the DHIS2 electronic transition. Recent sub-Saharan literature confirms that despite the introduction of molecular diagnostics, the underlying structural bottlenecks identified here namely workforce shortages, fragmented supervision, and supply chain fragility persist as modern barriers [35]. This study provides the empirical baseline necessary to understand that introducing new technology without addressing core health system determinants yields suboptimal returns.

### 4.3 The five-pillar framework for policy

To translate these findings into scalable strategies aligned with the WHO End TB Strategy and SDG 3.8, we propose an integrated, system-level framework [1]:

1. *Diagnostic Strengthening:* Scale molecular diagnostics and AI-assisted digital radiography across the PHCU tier to reduce reliance on subjective clinical diagnosis and capture missing pediatric cohorts [26], [27].
2. *Community Systems:* Revitalize the HEP by implementing targeted pediatric case-finding toolkits and continuous, hands-on clinical training for HEWs [20], [28].
3. *Supply Chain Reform:* Transition to decentralized buffer stock management policies and pilot blockchain technology to guarantee end-to-end transparency and prevent medication stockouts [29], [30], [31].
4. *Digital Health:* Deploy localized mHealth applications to facilitate remote adherence monitoring and digital default tracing, mitigating human resource shortages [32].
5. *Governance Improvements:* Decentralize healthcare decision-making to regional levels to mandate, fund, and enforce a minimum 90% compliance rate for quarterly supportive facility supervision [11], [25].

### 4.4 Limitations

The primary limitation is that data from 2010 to 2011 do not capture the contemporary post-GeneXpert landscape. However, as noted, recent evaluations verify that the structural bottlenecks regarding workforce density and logistics remain highly relevant [35]. Additionally, the cross-sectional elements of the facility audit limit our ability to make direct causal inferences.

## 5 Conclusion

This study documents significant, systematic gaps in pediatric tuberculosis detection and treatment success within primary health care units in Ethiopia. Grounded in implementation science and system frameworks, our proposed “Five-Pillar Framework” offers an empirical, scalable pathway to eliminate structural bottlenecks, optimize infectious disease programs, and advance universal health coverage (UHC) across Ethiopia and similar low- and middle-income countries.

## CRediT authorship contribution statement

**Dagmawi Mengesha:** Writing – review & editing, Writing – original draft, Visualization, Validation, Supervision, Software, Resources, Project administration, Methodology, Investigation, Funding acquisition, Formal analysis, Data curation, Conceptualization.

## Ethical approval

Secondary analysis approved by the Institutional Ethics Committee in January 2025 (Ref: ETH-2025-001).

## Declaration of competing interest

The authors declare that they have no known competing financial interests or personal relationships that could have appeared to influence the work reported in this paper.
